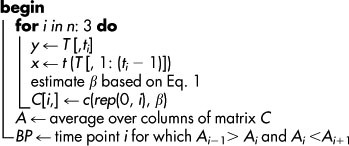# Segmentation of biological multivariate time-series data

**DOI:** 10.1038/srep08937

**Published:** 2015-03-11

**Authors:** Nooshin Omranian, Bernd Mueller-Roeber, Zoran Nikoloski

**Affiliations:** 1 Department of Molecular Biology, University of Potsdam, Karl-Liebknecht-Str. 24-25, Haus 20, 14476 Potsdam, Germany; 2 Systems Biology and Mathematical Modelling Group, Max Planck Institute for Molecular Plant Physiology, Am Muehlenberg 1, 14476 Potsdam, Germany

## Abstract

Time-series data from multicomponent systems capture the dynamics of the ongoing processes and reflect the interactions between the components. The progression of processes in such systems usually involves check-points and events at which the relationships between the components are altered in response to stimuli. Detecting these events together with the implicated components can help understand the temporal aspects of complex biological systems. Here we propose a regularized regression-based approach for identifying breakpoints and corresponding segments from multivariate time-series data. In combination with techniques from clustering, the approach also allows estimating the significance of the determined breakpoints as well as the key components implicated in the emergence of the breakpoints. Comparative analysis with the existing alternatives demonstrates the power of the approach to identify biologically meaningful breakpoints in diverse time-resolved transcriptomics data sets from the yeast *Saccharomyces cerevisiae* and the diatom *Thalassiosira pseudonana*.

Time-series data gathered from biological and technological systems capture the underlying dynamics of the ongoing processes. For a single component of the system, the corresponding time-series can be partitioned into intervals with predominant trends, e.g., increasing or decreasing^1^. The identified breakpoints are usually associated with major events contributing to the behavior of the components^2^. However, determining consensus intervals over multiple observed components of a given system, referred to as *multivariate time-series segmentation* (MTS-seg), remains a challenging computational problem^3^. MTS-seg has wide applications in computational systems biology^4^,^5^, market analysis^6^, and process control^7^.

In a multicomponent system, the progression of processes is a result of interactions among the components whose dependencies are reflected in the correlation structure of the respective data read-outs. For instance, in a biological system, the components include genes, proteins, and metabolites, whose changes can be monitored with high-throughput technologies^8^. Moreover, changes in the behavior of system's components may cause shifts in the correlation structure. MTS-seg can therefore be applied on time-resolved biological data to detect major changes as breakpoints in the systems behavior based on the temporal correlation structure.

Various approaches have been developed for the MTS-seg problem^9^,^10^,^11^, which can be categorized into four classes based on the computational methodology used: (1) clustering^4^,^12^,^13^,^14^, (2) graphical models^15^,^16^,^17^,^18^,^19^,^20^, (3) genetic algorithms^21^,^22^,^23^, and (4) regression.

While the first three categories have been well-investigated (see Ref. 5 for further details), the regression-based approaches provide a novel strategy for addressing the MTS problem, especially if regularization techniques (*e.g.*, least absolute shrinkage and selector operator (LASSO)^24^) are considered^25^. The regression-based approaches must account for the realistic scenario with a small number of time points (*n*) and large number of variables (*p*), typically arising in biological settings. For instance, Davis *et al.*^26^ applied minimum description length to detect the best fitting autoregressive (AR) model for each segment. This approach was recently extended in Ref. 23, where each segment is represented by a piecewise quantile regression model penalized for description length. Another regression-based approach addresses the MTS-seg problem by applying piecewise constant function on the MTS data^27^. The breakpoints are estimated by using total variation penalty while small jumps from the zero-mean are discarded. This method has also been extended to solve the MTS-seg problem by reformulating it as group LASSO regression^28^.

A further approach uses a discrete hidden logistic process which allows for smooth or abrupt changes in polynomial regression models. This method was first suggested to solve the problem of univariate time series segmentation/clustering^29^,^30^ which was then extended to multivariate time-series data sets^31^. This method uses an expectation-maximization algorithm to estimate the model parameters in an unsupervised fashion; however, the presented formulation of this approach relies on a pre-specified number of latent processes. Another recent approach that relies on dynamic programming is based on the simple piecewise polynomial regression mixture^32^; however, this approach may result in discontinuous segmentation, which is not meaningful in the analysis of time-series data from biological systems.

Finally, Preuß *et al.*^33^ introduced a nonparametric approach to infer breakpoints in the autoco-variance structure of the multivariate piecewise stationary process which relies on comparison of spectral distribution on different segments.

Despite these recent developments, however, the existing regression-based approaches suffer from several shortcomings related to the applicability on large data sets and the necessity to *a priori* specification of the breakpoints in the system. Moreover, most of these approaches are designed and applicable only for the case where components of the system are independent, which does not hold true in biological applications^34^. Our contribution is threefold: First we propose a formulation of the MTS-seg problem based on a fused LASSO regression, whereby the natural order of time points (features) is imposed in the fusion. We then propose a novel method to estimate the significance of the determined breakpoints which relies on clustering within each of the detected segments. Finally, we identify the key components for the determined segments, based on quantifying the effect that the removal of a set of components has on the established segments. To this end, we employ two criteria: structural and ontology-based homogeneities. We extensively illustrate the biological relevance of the proposed method through a comparative case study with the state-of-the-art alternative methods on transcriptomics MTS data from various experimental scenarios on the yeast *Saccharomyces cerevisiae* and the diatom *Thalassiosira pseudonana*.

## Results

### Fused LASSO formulation of the MTS-seg problem

We formulate the approach for the MTS-seg problem by using fused LASSO to consider the inherent order of time points. Therefore, each variable, corresponding to a time point, is described by a vector over the considered components. Since the relationship between variables changes at a breakpoint, it is expected that the variables for the preceding time points have negligible explanatory power in the regression model for the breakpoint variable; analogously, a breakpoint variable is expected to have small explanatory contribution for the time points following it.

Therefore, in a regression setting with a given variable (time point) as a response and the variables corresponding to the preceding time points as regressors, the breakpoint variable is expected to have zero regression coefficient, provided that all variables are scaled and centered. This idea can be readily captured by the fused LASSO formulation: Given time-series for *m* variables over *n* time points, 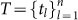, represented by a data matrix *M_m_*_×*n*_, we aim at determining a model for partitioning the time-series into *k* non-overlapping contiguous segments 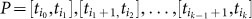, 1 ≤ *i_j_* < *i_j_*_+1_ ≤ *n*, 0 ≤ *j* < *k*, that span the whole series, *i.e.*, *i*_0_ = 1 and *i_k_* = *n*. Let *y_t_res__* be the profile of time point *t_res_*, (3 ≤ *res* ≤ *n*), and let the matrix 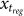 includes the profiles of all subsequent time points *t_reg_*, (*reg* ∈ [1, *res*)) which will be used as regressors. The fused LASSO is then given by:
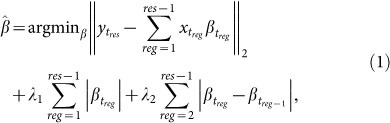
with *β* as a vector of regression coefficients. Solving the fused LASSO with every time point as a response, we obtain a lower-triangular matrix *C_n_*_×*n*_ containing the regression coefficients of the *n* models.

Breakpoints are then determined by examining the sequence *A* obtained by averaging the absolute values from each column of the matrix *C*. This sequence *A* summarizes the overall behavior and temporal patterns of the components over the examine time domain. The breakpoints correspond to the local minima of *A*, given by the time point *i* for which *A_i_*_−1_ > *A_i_* and *A_i_* < *A_i_*_+1_.

The assumption is that each segment captures a specific trend in the system's behavior. Therefore, the data profiles of the time points in a given segment should be similarly explained by the preceding time points. As a result, the breakpoints are those with weak relation. To this end, the “fusion” helps detect the consecutive time points, preceding the time point corresponding to the response, which can similarly explain the behavior of the response time point. The similarity of the explanatory behavior is captured by the closeness of the respective coefficients, assessed by the respective absolute value of their difference. While the LASSO constraint helps identify the likely true dependencies between regressors and the response (in case of large time-series data), the fusion penalty contributes to minimizing the differences of the coefficients (capturing the dependencies) between successive time points.

### Estimating the statistical significance of the segments

Segmentation constraints require that segments are internally homogeneous and externally heterogeneous with respect to the behavior of the components. These constraints can be readily examined by considering the quality of the clusters extracted from the different segments by using cluster quality indices^35^. The significance level of the resulting segmentation can be estimated by permutation testing in the following fashion: First, the segmentation approach is applied on the original data set and the average cluster quality index of choice is estimated over the resulting segments, denoted by *S*_0_:
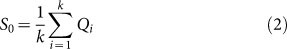
where *k* is the number of clusters and *Q_i_* is the quality index of the *i*'th cluster (segment). Then, the time points are randomly permuted and the segmentation approach is applied on the permuted data set. The resulting breakpoints are then used to segment the original data set. The empirical *p*-value is obtained as:
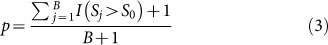
where *S_j_* is calculated using Eq. 2 with the results obtained from the *j*'th permutation, *I* is the indicator function, and *B* is the number of permutations.

### Determining key components

The breakpoints partition the time-series data into the sequence of segments. Each segment can reveal information about the temporal behavior of the system.

Given a set of segments 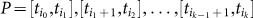 for which 1 ≤ *i_j_* < *i_j_*_+1_ ≤ *n*, 0 ≤ *j* < *k* while *i*_0_ = 1 and *i_k_* = *n*, next we introduce an approach to discover the key components for each segment which may be responsible for the breakpoints. To this end, we rely on the idea that a component is considered key if the removal of its time-series disturbs the estimated breakpoints on the entire data set. Determining the key components can be obtained by considering two criteria:

- *Structural homogeneity*: This criterion can be applied in cases that variables in the time-series data are not well characterized (annotated). In this case, the following steps are repeated for all except the last segment in *P* (Algorithm 1). First, the data profiles located in the time interval corresponding to the segment 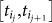 are clustered by using an algorithm of choice (the partitioning around medoid (pam) clustering is used in this analysis^36^). Then, the profiles of the components in each cluster are iteratively removed, and the segmentation is determined on the remaining data profiles, followed by inspection of any change in the breakpoints. To determine the maximum number of key components in a feasible manner, the clustering is performed starting with *l* = 2 clusters. The number of clusters, *l*, is increased by one if none of the *l* clusters affected the breakpoints. The clustering procedure is repeated until at least one cluster is found to be the key for the segmentation or the number of clusters equals the number of components.
- *Ontology-based homogeneity*: Biological data can be grouped based on conceptual features given in an ontology. For example, in case of transcriptomics data, genes can be grouped based on the pathways or biological processes in which they participate. Therefore, an analogous approach as for the structural homogeneity can be applied. The divisive step can be readily applied due to the hierarchical nature of the existing ontologies^37^. More specifically, to cluster the profiles of genes based on their biological homogeneity, we proceeded as follows: (1) The GO terms were obtained for the genes to be clustered. (2) The *k*means clustering was applied for the *k* = 2: 40 number of clusters. (3) For each *k*, the biological homogeneity of the clusters was estimated using biological homogeneity index^38^. (4) The value for *k* which was associated with the maximum biological homogeneity index was stored. (5) The procedure was repeated 1000 times starting from step (2). (6) The histogram of the obtained values for *k* from the 1000 runs was used to determine the most frequent value of *k* which resulted in the highest biological homogeneity for the clusters.

### Applications of the approach

We applied the proposed approach for MTS-seg problem based on fused LASSO regression to several data sets, including: synthetic, yeast's metabolic and cell cycles, and *Thalassiosira pseudonana*'s diel growth state transition. Moreover, we compared its performance with the other regression-based approach intended for solving the MTS-seg problem.

#### Synthetic data

**Figure 1:**
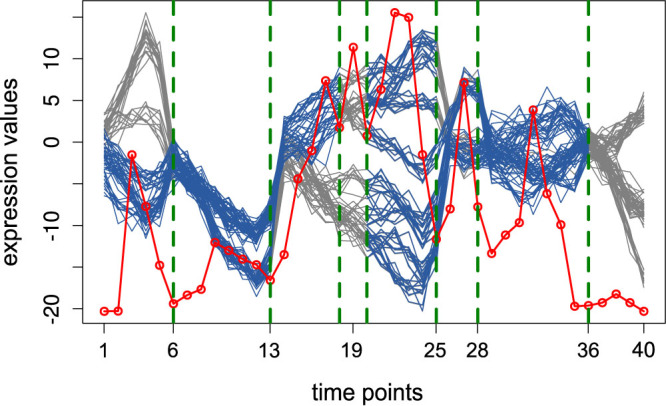
Segmentation over synthetic data. The green dashed lines show the obtained breakpoints. The blue colored curves at each segment illustrate the respective key components. The gray colored parts of the time-series denote the variables not involved in the local changes at the corresponding breakpoints. The red dots, connected by a red line, represent the sequence *A* (column-averages of the absolute values of the regression coefficients in the matrix *C*).

To investigate the performance of the algorithm, we created synthetic time-series data for 80 variables over 40 time points (see Figure 1 and Methods). The segmentation points corresponded to time points 5, 13, 25, 28 and 35. Figure 1 illustrates the segments obtained by the proposed approach with the *p–value* of 0.04 for 1000 permutations. The synthetic MTS data are segmented into 8 segments with breakpoints at 6, 13, 18, 20, 25, 29, and 35. All real breakpoints were captured, and only two additional breakpoints were included. This was likely due to the apparent change in the behavior of the time-series between the time points 13 and 25, which was not controlled in the generation of the data (see Methods).

In contrast, the group fused LASSO approach from Bleakley *et al.*^27^ on the same data resulted in the five segments with following breakpoints: 6, 12, 16, and 24. However, this approach could not detect the late breakpoints at 28 and 35. The extensive comparison of the contending methods with our approach is given in the Supplementary information S1 (including Fig. S1 and Table S1).

The time-series data were next structurally clustered in each segment to obtain the key components. The colored curves at each segment show the key components which led to a structural change at the specified breakpoint. Since detection of key components is based on clustering, only the segments with more than two time points were inspected for the key components.

#### Yeast's metabolic and cell cycles

Motivated by the predictions from applying the approach on the synthetic data set, we next investigated the MTS-seg on the transcriptomics data sets from yeast metabolic cycle^39^, cell cycle^40^, and the experiment capturing the effect of oxidative stress, induced by hydrogen peroxide (HP) treatment, on the yeast's cell cycle^41^. In all data sets, we filtered out the genes which: (1) contain missing values, (2) have no gene ontology (GO) annotation, and (3) their coefficients of variation are smaller than 1. We focused on yeast's metabolic cycle, and the results for the other data sets as well as the comparison with other methods are detailed in the Supplementary Information S1 (including Fig. S2–S4 and Table S2–S4).

The yeast metabolic cycle (YMC) consists of the following three successive phases spanning each ~5 h: (1) a reductive charging (R/C) phase, involving non-respiratory metabolism (glycolysis and fatty acid oxidation) and protein degradation, (2) oxidative metabolism (Ox), in which respiratory processes are used to generate adenosine triposphate (ATP), (3) reductive metabolism (R/B), marked by a decrease in oxygen uptake and dominance of DNA replication, mitochondrial biogenesis, ribosome biogenesis, and cell division^39^. The data set included the time-resolved expression of 6555 genes (with 9335 microarray probes) over 36 time points (separated by ~25-min intervals) over three consecutive cell cycles. Clustering of the obtained transcript profiles was employed in Tu *et al.*^39^ to show that YMC controls the timing of key cellular and metabolic processes to allow coordination of anabolic and catabolic processes for efficient energy production and usage. Therefore, this data set can serve as a benchmark for testing our proposed algorithms for the MTS-seg problem.

With the filtering steps mentioned above, the number of genes was reduced from 6555 to 255. The latter were employed to determine the segmentation based on the proposed approach. Due to the presence of recurrent changes on the global level, two segmentation points, at 12–13 and 24–25 should be detected. These breakpoints delineate the three considered cell cycles. In addition, due to the presence of the alternation phases in the metabolic cycle, each of the three cycles should contain at least one more segmentation point.

**Figure 2:**
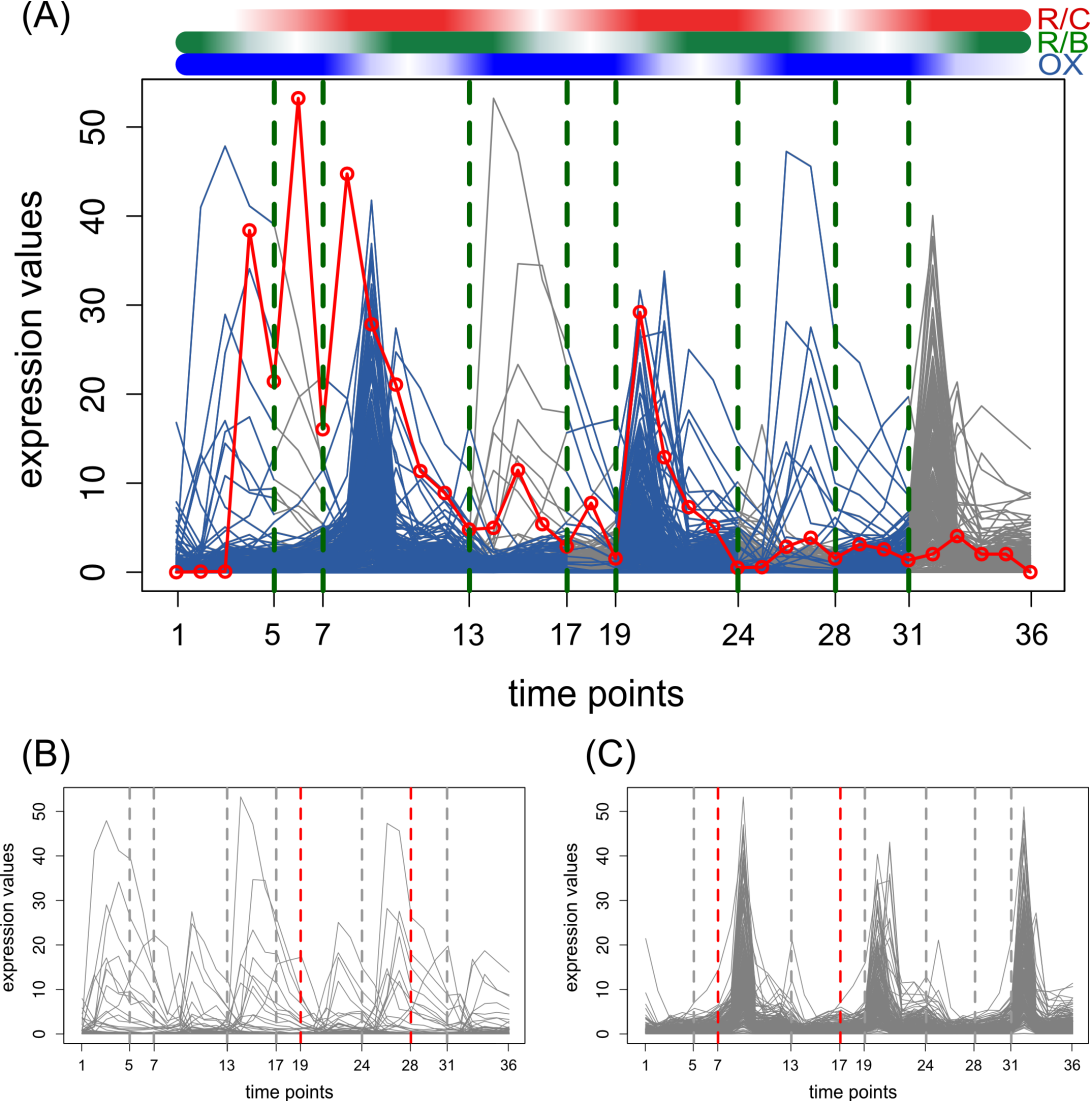
Segmentation over yeast's metabolic cycle. (A) The expression profiles of 255 genes over 36 time points (separated by ~25-min intervals) over three consecutive cell cycles. The green dashed lines denote the obtained breakpoints. The blue colored curves at each segment illustrate the key components. The gray colored parts of the time-series denote the variables not involved in the local changes at the corresponding breakpoints. The red dots, connected by a red line, represent the sequence *A* (column-averages of the absolute values of the regression coefficients in the matrix *C*). Genes were grouped into two clusters (B) and (C) based on their biological homogeneity. (B) includes 41 genes which are predominantly involved in M, G1, and G1/S phases and (C) contains 214 genes related to the metabolic processes. Each cluster is specifically responsible for the breakpoints represented by red dashed lines.

Applying the proposed approach on YMC time-series data resulted in 9 segments which was significant at the level of 0.088. Figure 2.A illustrated the segmentation and the Supplementary Table S2 compares the segmentation results with the previous studies^4^,^5^,^27^. As it is shown in the Figure 2.A, the starting points of the three aforementioned cycles were captured by this approach. The inferred breakpoints are at the 5, 7, 13, 17, 19, 24, 28 and 31 time points, and the breakpoints delineating the three considered cell cycles (time points 13 and 24) could be precisely detected. The approach of Bleakley *et al.*^27^ failed to infer all three cycles, as well as the other breakpoints in each cycle (breakpoints at 8, 9, 26, 31, and 32).

We used biological homogeneity index to cluster the genes based on the biological process (BP). Two clusters were generated as a result, including 41 and 214 genes, respectively. Genes in the first cluster were predominantly involved in the M, G1, and G1/S phases of the cell cycle (Figure 2.B), while genes in the second cluster were related to the metabolic processes (Figure 2.C), as determined by gene enrichment analysis (Supplementary Table S5).

In Figures 2.B and 2.C, the red dashed lines visualize the points at which the specific cluster highly contributed to the inferred breakpoints. Considering the third cycle (time interval between 25 h and 36 h), genes that were involved in M, G1, and G1/S phases were identified to contribute to the detection of a breakpoint at time point 28 h, while the genes involved in metabolic processes were involved at the breakpoint at 31 h.

#### Diel growth state transition of diatom *Thalassiosira pseudonana*

**Figure 3:**
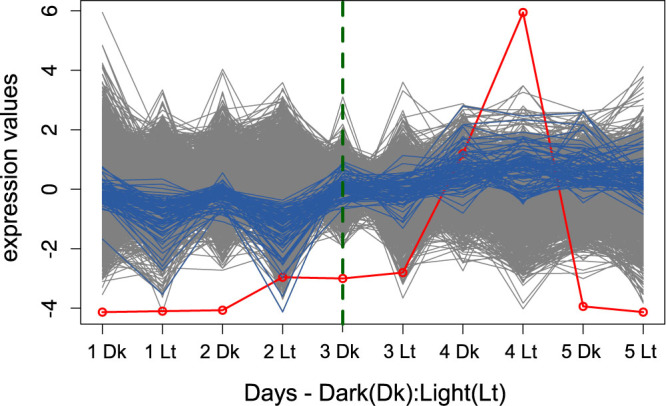
Segmentation over diel growth state transition of diatom *Thalassiosira pseudonana*. The expression profile of 5417 genes illustrated over cycles of 12:12 h dark(Dk):light(Lt) in 5 days. The green dashed line shows the obtained breakpoint. The blue colored curves illustrate the key components within the first segment which led to the only structural changes at [3 Dk]. The gray colored parts of the time-series denote the variables that were not involve in the local changes at the corresponding breakpoints, as detected by the approach. The red dots, connected by a red line, represent the sequence *A* (column-averages of the absolute values of the regression coefficients in the matrix *C*).

The proposed approach is also applicable to short time-series data. To illustrate this application, we investigated the MTS-seg on transcriptomics MTS data from *Thalassiosira pseudonana*'s diel growth state transition^42^. Ashworth *et al.*^42^ measured the transcript level of *Thalassiosira pseudonana* in five days on a 12:12 h dark(Dk):light(Lt) cycle to find the key regulators responsible for exponential and stationary phase modulation as well as diel phase reversal (Fig. 3). They reported a major shift on the third day between 12 h dark and 12 h light (3 Dk and 3 Lt). Up to the third day 12 h light (3 Lt), genes labeled as “exponential” showed higher expression than genes labeled as “stationary”; however, from the third day light (3 Lt) on, the stationary genes showed higher expression. Consequently, the time between 3 Dk and 3 Lt could be considered as a transition period due to the shift between exponential and stationary phase causing major change in gene expression levels.

The proposed MTS segmentation approach applied on the transcription profiles of 5417 genes (with the coefficient of variation above 0.3) over 10 time points resulted in 2 segments with breakpoint at 3 Dk with the significance level of 0.14 illustrated in Figure 3.

Next, we used three groups of genes annotated with exponential and stationary phase modulation as well as diel phase reversal (Supplementary Table S7 obtained from Supplementary Dataset1 in Ref. 42). However, the removal of all these genes did not affect the result of the clustering which indicates further genes that also affect the shift between 3 Dk and 3 Lt. Therefore, and due to the lack of GO annotation for *Thalassiosira pseudonana*, we used structural clustering in order to detect the key components. We could obtain 82 genes as key components whose behavior led to the break point at 3 Dk (Supplementary Table S6). The profile of these genes are highlighted in blue in Figure 3. Among the key components 9 genes were in the list of genes which were more highly expressed at dawn during the exponential and diel phase, based on the Supplementary Dataset1 from Ref. 42.

## Discussion

Here we introduced a regression formulation of the MTS-seg problem based on fused LASSO. The breakpoints were determined by inspecting the changes in the regression coefficients over a series of regression models. In addition, we proposed a method to determine the statistical significance analysis of the inferred breakpoints by applying a cluster-based approach and a cluster quality index of choice.

We note that all findings on the statistical significance of the found breakpoints were obtained based on the cluster quality measure. Due to the difference in behavior of different cluster quality indices^43^, the determined *p*-values should be carefully interpreted based on the properties of the investigated data sets. Nevertheless, the proposed procedure provides a general method to couple clustering in segments with the determined breakpoints with the aim of establishing the significance of the latter.

Moreover, we devised a clustering-based approach to identify the key components giving rise to the abrupt changes in the system. We could identify the order of processes for a metabolic cell cycle from yeast data set. In addition, application of this approach to diel growth state transition of diatom result in a group of key components which are highly expressed at dawn during exponential and diel phase. This approach elaborate on biological aspect of the dynamic relationships underlying biological processes.

Applying the method to different data sets supported the reliability and significance of the determined breakpoints in the well-documented cases of yeast's cell and metabolic cycles. Unlike other approaches, we did not impose restriction on the number of time points included in each segment, rendering our method applicable to short time-series typical in biological studies. In addition, the comparative analysis demonstrated that the regression-based approach performs better in comparison to the state-of-the-art algorithm.

Improvement to the proposed approach can be obtained by imposing constraints to the fused LASSO regression such as in the recent study from Sue and Tibshirani *et al.*^44^. To further investigate on the accuracy and sparsity of the breakpoint set, various constraints can be imposed to the fused LASSO. For instance, if a time point as a regressor does not have explanatory power for the response time point, the regression coefficients of all preceding time points can be neglected.

Formulation of the segmentation with additional constraints, including different segmentations for groups of entities, would render our approach widely applicable in different fields.

## Methods

### Segmentation algorithm

The segmentation algorithm, given in Algorithm 2, is implemented in R [http://www.R-project.org] by using the package penalized [http://cran.r-project.org/web/packages/penalized]). The regression coefficients were robustly estimated by *K*-fold cross validation (K selected based on the available time points) together with the optimal values for *λ*_1_ and *λ*_2_ from the range [1,50]. We assumed that no breakpoints could occur in the first and the last three time points of the time-series, as robust changes could only be detected after at least 4 consecutive time points^5^. The implementation is available at http://mathbiol.mpimp-golm.mpg.de/Segmentation-fLASSO/index.html.

### Significance and key components of segmentation

In the current implementation, the significance level of the segmentation was based on the average silhouette width^45^. To determine the structural homogeneity, partitioning around medoids algorithm (pam) function in the R package cluster [http://stat.ethz.ch/R-manual/R-patched/library/cluster/html/pam.html] with Pearson correlation was employed to cluster data profiles. For biological data, the ontology-based homogeneity was inspected based on the biological process (BP) category of the Gene Ontology^46^. The GO enrichment analysis for each cluster was performed using hypergeometric test for which we used phyper function from R package stats [https://stat.ethz.ch/R-manual/R-patched/library/stats/html/Hypergeometric.html] and annotation package ygs98.db [http://www.bioconductor.org/packages/release/data/annotation/html/ygs98.db.html].

### Synthetic data set

To create these segmentation points, a number of data profiles were generated for each segment by simulating a zero-mean autoregressive moving average (ARIMA) model (described in Ref. 5). The number of profiles simulated for the six segments, [1–5], [6–13], [14–25], [26–28], [29–35] and [36–40], was set to 5, 2, 8, 3, 7 and 4, respectively. Each of the 80 variables was obtained by randomly sampling a characteristic data profile in each segment. In addition, a normally distributed error term, *N*(0, 1), was added to the sampled profile value at each time point. The code for generating synthetic data is available at http://mathbiol.mpimp-golm.mpg.de/Segmentation-fLASSO/index.html.

## Supplementary Material

Supplementary Information Supplementary information

## Figures and Tables

**Table 1:** 

**Algorithm 1:** Key components detection.
** Data**: *T* time-series data with *n* time points
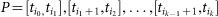
** Result**: *key*, list of key components for each breakpoint
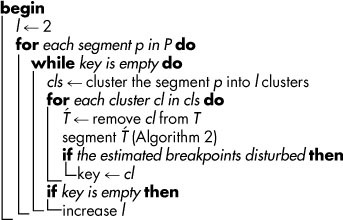

**Table 2:** 

**Algorithm 2:** Regularized segmentation.
** Data**: *T* time-series data with *n* time points
** Result**: *BP*, breakpoints