# Supplementary material for: Segmentation of biological multivariate time-series data
Source: Sci Rep. 2015 Mar 11;5:8937. doi: 10.1038/srep08937 (PMC5390911; doi:10.1038/srep08937)
Supplement: Supplementary Information [file srep08937-s1.pdf]

Supplementary information S1
Segmentation of biological multivariate time-series data

Nooshin Omranian[1,2], Bernd Mueller-Roeber[1], Zoran Nikoloski[2,*]
[1] Department of Molecular Biology, University of Potsdam, Karl-Liebknecht-Str. 24-25, Haus 20, 14476 Potsdam, Germany
[2] Systems Biology and Mathematical Modelling Group, Max Planck Institute for Molecular Plant Physiology, Am Muehlenberg 1, 14476 Potsdam, Germany
*** E-mail: nikoloski@mpimp-golm.mpg.de**

Synthetic data

In this section, we provide additional elaboration of the results from different approaches applied on the synthetic data, described in the main text in Omranian *et al.* (2013). The results are succinctly summarized in Table S1, and Figure S1 showing the comparison between the obtained segmentations. The actual breakpoints are 7, 12, and 21.

Yeast's metabolic cycle

In this section, we provide additional elaboration of the results from different approaches applied on the yeast's metabolic cycle (YMC) data, described in the main text. The results are succinctly summarized in Table S2, showing the comparison between the obtained segmentations.

Yeast's cell cycle

In this section, we provide additional elaboration of the results from different approaches applied on the yeast's cell cycle (YCC) data. With the filtering step, the number of genes was reduced from 6076 to 2071. The characteristics of the resulting segmentations are summarized in Table S3 and Figure S3. Based on the work done by Spellman *et al.* (1998), the data should be segmented into 5 segments representing 5 cycles for which each cycle includes the following phases: M/G1, G1, S, G2, and M. Each of the M/G1, G1 and S phases lasts 2 time points while the G2 phase lasts only one time point, as described in Ramakrishnan *et al.* (2010). Therefore, as shown in Table S3, our method revealed the cell cycles in the YCC data.

Oxidative stress and yeast's cell cycle

In this section, we provide additional elaboration of the results from the different approaches applied on the data capturing the effect of oxidative stress, induced by hydrogen peroxide (HP), on the yeast's cell cycle. With the filtering step, the number of genes was reduced from 4771 to 1189.

The characteristics of the resulting segmentations are summarized in Table S4 and Figure S4. Based on the work done by Shapira *et al.* (2004), we could capture all phases in the system which correspond to the G1, S, G2, G2/M phases of the cell cycle.

Figure Legends

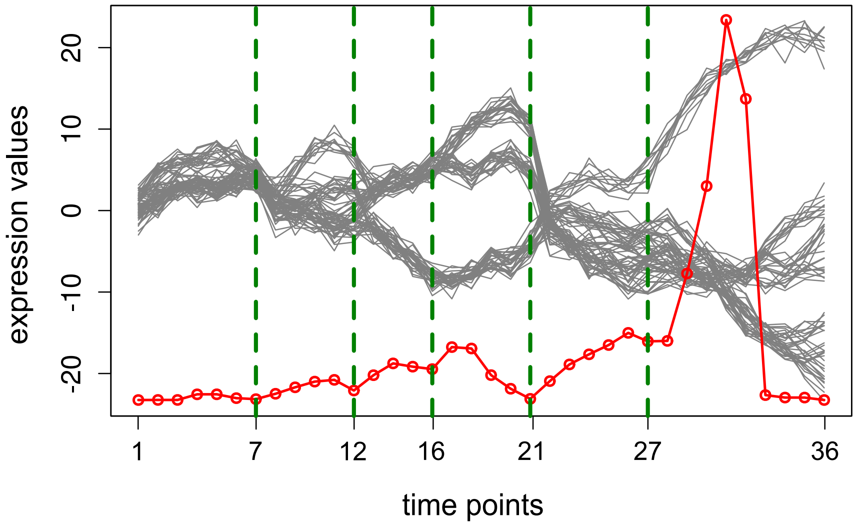

Figure S1. Segmentation over synthetic data from Omranian *et al.* (2013). The green dashed lines show the obtained breakpoints. The red dots, connected by a red line, represent the sequence A (column-averages of the absolute values of the regression coefficients in the matrix C).

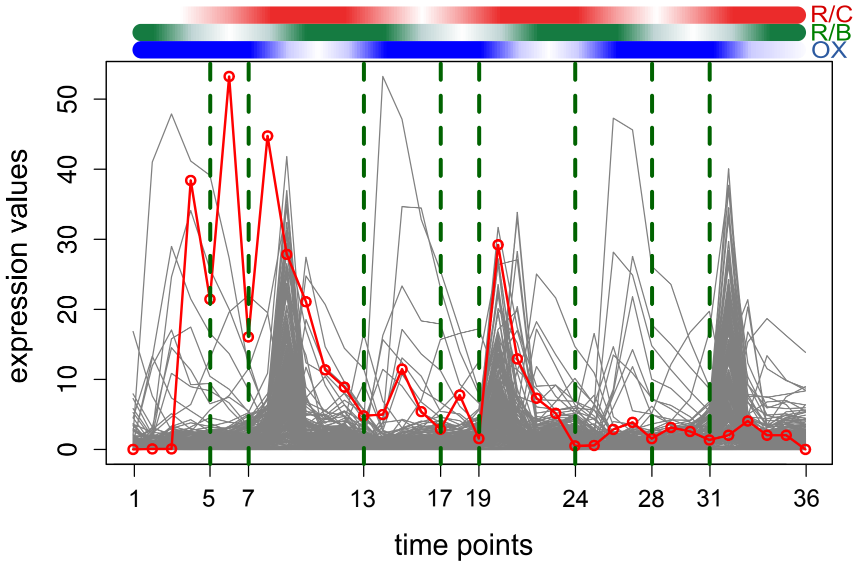

Figure S2. Segmentation over Yeast's metabolic cycle. The expression profile of 255 genes illustrated over 36 time points (separated by $\sim$ 25-min intervals) over three consecutive cell cycles. The green dashed lines show the obtained breakpoints. The red dots, connected by a red line, represent the sequence A (column-averages of the absolute values of the regression coefficients in the matrix C).

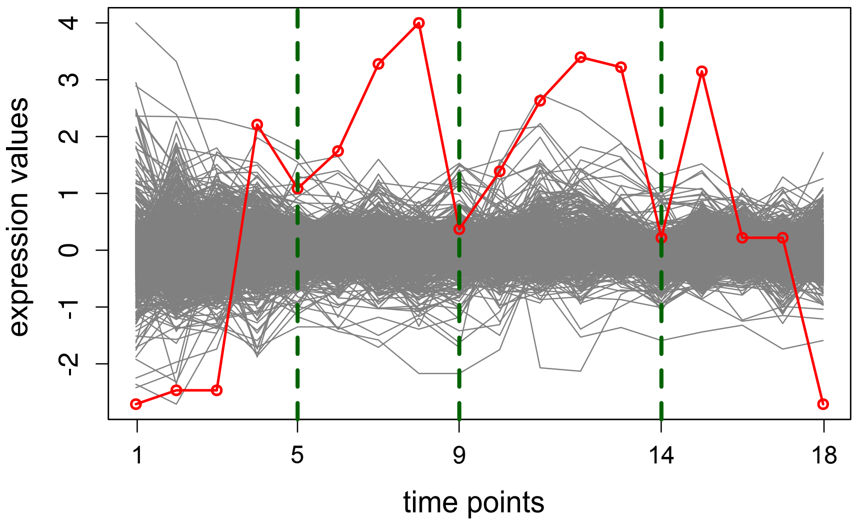

Figure S3. Segmentation over Yeast's cell cycle. The green dashed lines show the obtained breakpoints. The red dots, connected by a red line, represent the sequence A (column-averages of the absolute values of the regression coefficients in the matrix C).

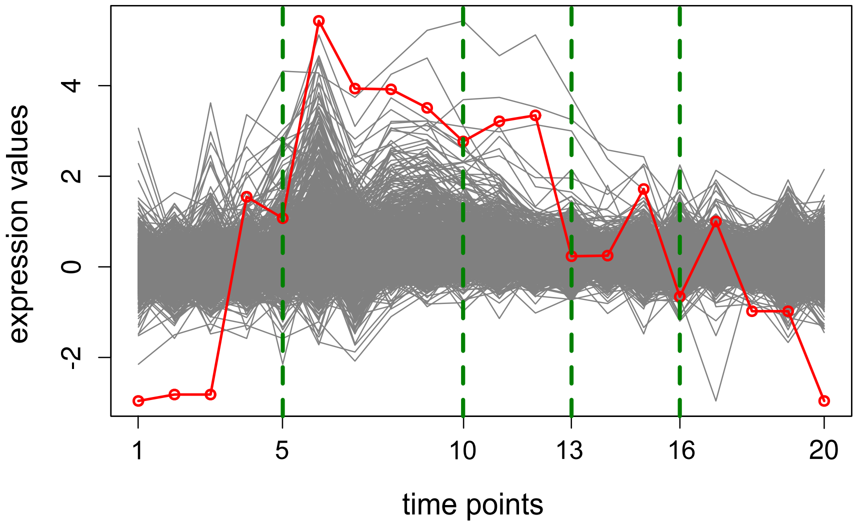

Figure S4. Segmentation over Yeast's cell cycle. The green dashed lines show the obtained breakpoints. The red dots, connected by a red line, represent the sequence A (column-averages of the absolute values of the regression coefficients in the matrix C).

Tables

Table S1. Optimal segmentation for synthetic data. The first part of the table comprises the result of the optimal segmentation for synthetic data based on the regularized regression approach implemented in Algorithm 1. The second part includes the result based on Bleakley *et al.* (2011) approach. The third and the forth parts show the results based on the method of Omranian *et al.* (2013), penalized longest path algorithm using number of segments and distribution of length of the segments to calculate the penalty of a path, respectively. The lower part contains the result based on the method of Ramakrishnan *et al.* (2010). The upper part of the table includes the number of segments k, breakpoints, and tuning parameters corresponding to fused LASSO regularization parameters, λ_1 and λ_2. In the third part of the table, the first and second columns show the name and the type of network properties used to determine the distances: G stands for global, L for local, and LG for local-global. The third column includes the number k for each of the three methods and the resulting segments are given in the forth column. The fifth and sixth columns in the third and forth parts present the values of lower (ν_{min}) and upper (ν_{max}) bound of the tuning parameter ν with dynamic programming approach. The lower part also includes minimum and maximum length of the segments, i.e., l_{min} and l_{max}, as parameters of the contending method.

**Algorithm 1**		k	**Breakpoints**	λ_1	λ_2
		6	7, 12, 16, 21 and 27	[1,50]	[1,50]
**Bleakley *et al.* (2011)**		k	**Breakpoints**		
		5	13, 21, 28 and 32		
**Omranian *et al.* Penalized (number)**	**Type**	k	**Breakpoints**	ν_{min}	ν_{max}
relative density	G	5	4, 8, 12 and 21	0.05	6.00
degree	L	5	4, 8, 12 and 24	1.50	11.13
closeness	LG	4	4, 15 and 20	0.05	4.47
betweenness	LG	7	4, 8, 12, 17, 21 and 29	1.06	12.10
**Omranian *et al.* Penalized (length)**	**Type**	k	**Breakpoints**	ν_{min}	ν_{max}
relative density	G	9	4, 8, 12, 16, 20, 24, 28 and 32	0.05	6.00
degree	L	8	5, 9 ,13, 18, 23, 27 and 32	1.50	11.13
closeness	LG	4	4, 16 and 20	0.05	4.47
betweenness	LG	8	4, 9, 13, 17, 21, 25 and 30	1.06	12.10
**Ramakrishnan *et al.* (2010)**		k	**Breakpoints**	l_{min}	l_{max}
		6	5, 12, 19, 24 and 30	4	7
		5	7, 12, 21 and 29	4	9
		4	6, 12 and 24	4	12
		3	7 and 22	4	15
		3	7 and 25	4	20
		2	25	4	28
		2	7	4	32

Table S2. Optimal segmentation for yeast's metabolic cycle (YMC) data with the same preprocessing has been applied in Ramakrishnan *et al.* [15]. The first part of the table comprises the result of the optimal segmentation for synthetic data based on the regularized regression approach implemented in Algorithm 1. The second part includes the result based on Bleakley *et al.* (2011) approach. The third and the forth parts show the results based on the method of Omranian *et al.* (2013), penalized longest path algorithm using number of segments and distribution of length of the segments to calculate the penalty of a path, respectively. The lower part contains the result based on the method of Ramakrishnan *et al.* (2010). The upper part of the table includes the number of segments k, breakpoints, and tuning parameters corresponding to fused LASSO regularization parameters, λ_1 and λ_2. In the third part of the table, the first and second columns show the name and the type of network properties used to determine the distances: G stands for global, L for local, and LG for local-global. The third column includes the number k for each of the three methods and the resulting segments are given in the forth column. The fifth and sixth columns in the third and forth parts present the values of lower (ν_{min}) and upper (ν_{max}) bound of the tuning parameter ν with dynamic programming approach. The lower part also includes minimum and maximum length of the segments, i.e., l_{min} and l_{max}, as parameters of the contending method.

**Algorithm 1**		k	**Breakpoints**	λ_1	λ_2
		9	5, 7, 13, 17, 19, 24, 28 and 31	[1,50]	[1,50]
**Bleakley *et al.* (2011)**		k	**Breakpoints**		
		6	8, 9, 26, 31 and 32		
**Omranian *et al.* Penalized (number)**	**Type**	k	**Breakpoints**	ν_{min}	ν_{max}
relative density	G	6	4, 9, 13, 20 and 31	0.05	5.60
degree	L	8	4, 8, 12, 16, 20, 24 and 32	4.35	13.50
closeness	LG	6	4, 9, 17, 21 and 31	0.05	4.24
betweenness	LG	6	4, 8, 12, 20 and 24	3.80	14.52
**Ramakrishnan *et al.* [15]**		k	**Breakpoints**	l_{min}	l_{max}
		8	6, 10, 14, 18, 22, 26 and 31	4	7

Table S3. Optimal segmentation for yeast's cell cycle (YCC) data. The first part of the table comprises the result of the optimal segmentation for synthetic data based on the regularized regression approach implemented in Algorithm 1. The second part includes the result based on Bleakley *et al.* (2011) approach. The third and the forth parts show the results based on the method of Omranian *et al.* (2013), penalized longest path algorithm using number of segments and distribution of length of the segments to calculate the penalty of a path, respectively. The lower part contains the result based on the method of Ramakrishnan *et al.* (2010). The upper part of the table includes the number of segments k, breakpoints, and tuning parameters corresponding to fused LASSO regularization parameters, λ_1 and λ_2. In the third part of the table, the first and second columns show the name and the type of network properties used to determine the distances: G stands for global, L for local, and LG for local-global. The third column includes the number k for each of the three methods and the resulting segments are given in the forth column. The fifth and sixth columns in the third and forth parts present the values of lower (ν_{min}) and upper (ν_{max}) bound of the tuning parameter ν with dynamic programming approach. The lower part also includes minimum and maximum length of the segments, i.e., l_{min} and l_{max}, as parameters of the contending method.

**Algorithm 1**		k	**Breakpoints**	λ_1	λ_2
		4	4, 9 and 14	[1,50]	[1,50]
**Bleakley *et al.* (2011)**		k	**Breakpoints**		
		2	4		
**Omranian *et al.* Penalized (number)**	**Type**	k	**Breakpoints**	ν_{min}	ν_{max}
relative density	G	4	4, 8 and 12	0.05	4.08
degree	L	3	6 and 14	6.64	12.72
closeness	LG	3	5 and 14	0.05	5.64
betweenness	LG	4	4, 8 and 14	9.15	15.38
**Ramakrishnan *et al.* [15]**		k	**Breakpoints**	l_{min}	l_{max}
		6	3, 6, 9, 12 and 15	3	5

Table S4. Optimal segmentation for data from oxidative stress, induced by hydrogen peroxide (HP), on yeast's cell cycle. The first part of the table comprises the result of the optimal segmentation for synthetic data based on the regularized regression approach implemented in Algorithm 1. The second part includes the result based on Bleakley *et al.* (2011) approach. The third and the forth parts show the results based on the method of Omranian *et al.* (2013), penalized longest path algorithm using number of segments and distribution of length of the segments to calculate the penalty of a path, respectively. The lower part contains the result based on the method of Ramakrishnan *et al.* (2010). The upper part of the table includes the number of segments k, breakpoints, and tuning parameters corresponding to fused LASSO regularization parameters, λ_1 and λ_2. In the third part of the table, the first and second columns show the name and the type of network properties used to determine the distances: G stands for global, L for local, and LG for local-global. The third column includes the number k for each of the three methods and the resulting segments are given in the forth column. The fifth and sixth columns in the third and forth parts present the values of lower (ν_{min}) and upper (ν_{max}) bound of the tuning parameter ν with dynamic programming approach. The lower part also includes minimum and maximum length of the segments, i.e., l_{min} and l_{max}, as parameters of the contending method.

**Algorithm 1**		k	**Breakpoints**	λ_1	λ_2
		5	5, 10, 13 and 16	[1,50]	[1,50]
**Bleakley *et al.* (2011)**		k	**Breakpoints**		
		4	5, 10 and 15		
**Omranian *et al.* Penalized (number)**	**Type**	k	**Breakpoints**	ν_{min}	ν_{max}
relative density	G	3	4 and 15	0.05	4.43
degree	L	3	5 and 15	5.78	12.60
closeness	LG	4	4, 8 and 16	0.05	5.71
betweenness	LG	4	5, 9 and 13	8.43	15.11
**Ramakrishnan *et al.* [15]**		k	**Breakpoints**	l_{min}	l_{max}
		4	4, 11 and 14	3	7

Supplementary Table S5 - Over-enriched BP terms at significance level 0.05. The yeast's metabolic cell cycle transcription data, biologically clustered into two clusters. The result of GO enrichment analysis using biological process terms and significance level of 0.05 are

cluster 1 -

BP	P_value	GOTerm	Genes	Names
GO:0000003	2.39E-05	reproduction	4460_i_at;6459_at;7859_at	SAE3;MCD1;MEI5
GO:0000079	0.000371	regulation of cyclin-dependent protein kinase activity	5609_at;7993_at	DSE1;CLN2
GO:0000080	0.00993895	G1 phase of mitotic cell cycle	4899_at	CLB6
GO:0000082	0.02012023	G1/S transition of mitotic cell cycle	6337_at;9150_at;9683_at	SWI5;PCL1;YOX1
GO:0000087	0.0016603	M phase of mitotic cell cycle	4899_at;6641_at;7993_at;9150_at	CLB6;PCL9;CLN2;PCL1
GO:0000114	0.02012023	regulation of transcription involved in G1 phase of mitotic cell cycle	3628_f_at	COS10
GO:0000278	1.78E-06	mitotic cell cycle	7546_at	OPT2
GO:0000279	2.69E-07	M phase	6453_at;6455_s_at;7554_at	ENA5;ENA2;ARR3
GO:0000280	0.00993895	nuclear division	10164_at;10576_at;10755_at;11079_at;11256_at;11301_i_at;3628_f_at;3934_at;3989_at;4358_at;4392_at;4460_i_at;4787_at;4796_at;4899_at;5177_at;5609_at;5643_at;5655_n_at;6337_at;6453_at;6455_s_at;6459_at;6641_at;7204_at;7546_at;7554_at;7777_at;7859_at;7907_at;7912_at;7981_at;7993_at;8251_at;8716_s_at;8922_at;9150_at;9185_at;9203_at;9683_at;9712_at	TOS4;PRY2;PIR1;PRY3;IMA5;FLO1;COS10;RTM1;BI4;SPO16;DSE2;SAE3;TOS2;BNS1;CLB6;RCK1;DSE1;RNR1;MEI4;SWI5;ENA5;ENA2;MCD1;PCL9;AMN1;OPT2;ARR3;ECM23;MEI5;SVS1;AIM44;GRE1;CLN2;SFG1;AAD15;SUN4;PCL1;TOS6;EGT2;YOX1;AIM33
GO:0000707	0.02012023	meiotic DNA recombinase assembly	3989_at	BI4
GO:0000724	0.02012023	double-strand break repair via homologous recombination	4899_at	CLB6
GO:0000725	0.02012023	recombinational repair	6459_at	MCD1
GO:0000730	0.02012023	DNA recombinase assembly	7204_at	AMN1
GO:0000749	0.02012023	response to pheromone involved in conjugation with cellular fusion	6459_at	MCD1
GO:0000910	0.02691926	cytokinesis	6453_at;6455_s_at;7554_at	ENA5;ENA2;ARR3
GO:0000920	0.02012023	cytokinetic cell separation	6459_at;7204_at;9203_at	MCD1;AMN1;EGT2
GO:0001932	0.000371	regulation of protein phosphorylation	4358_at;6459_at;7204_at;7546_at;8922_at;9150_at;9203_at	SPO16;MCD1;AMN1;OPT2;SUN4;PCL1;EGT2
GO:0006259	0.0002024	DNA metabolic process	3989_at	BI4
GO:0006281	0.02235693	DNA repair	6459_at	MCD1
GO:0006302	0.00993895	double-strand break repair	6459_at	MCD1
GO:0006310	0.00026088	DNA recombination	6459_at	MCD1
GO:0006468	4.87E-05	protein phosphorylation	4358_at;4460_i_at;4787_at;4796_at;5177_at;5609_at;5655_n_at;6337_at;7859_at;7993_at	SPO16;SAE3;TOS2;BNS1;RCK1;DSE1;MEI4;SWI5;MEI5;CLN2
GO:0006754	0.02012023	ATP biosynthetic process	4358_at;4460_i_at;4796_at;5177_at;5655_n_at;6337_at;6459_at;7204_at;7859_at;9203_at	SPO16;SAE3;BNS1;RCK1;MEI4;SWI5;MCD1;AMN1;MEI5;EGT2
GO:0006793	0.01148149	phosphorus metabolic process	3989_at;4358_at;4460_i_at;4899_at;5643_at;5655_n_at;6337_at;6459_at;7859_at	BI4;SPO16;SAE3;CLB6;RNR1;MEI4;SWI5;MCD1;MEI5
GO:0006796	0.01148149	phosphate-containing compound metabolic process	4899_at;5177_at;5643_at;6453_at;6455_s_at;6641_at;7993_at;9150_at	CLB6;RCK1;RNR1;ENA5;ENA2;PCL9;CLN2;PCL1
GO:0006814	0.00993895	sodium ion transport	4899_at;5177_at;5643_at;6453_at;6455_s_at;6641_at;7993_at;9150_at	CLB6;RCK1;RNR1;ENA5;ENA2;PCL9;CLN2;PCL1
GO:0006869	0.02012023	lipid transport	7554_at	ARR3
GO:0006950	0.00488052	response to stress	8922_at	SUN4
GO:0006974	0.00445913	response to DNA damage stimulus	6459_at;7204_at;9203_at	MCD1;AMN1;EGT2
GO:0007049	4.18E-10	cell cycle	4787_at	TOS2
GO:0007067	0.00276299	mitosis	4392_at;7777_at;8251_at	DSE2;ECM23;SFG1
GO:0007109	0.02012023	cytokinesis, completion of separation	4899_at;5177_at;6641_at;7993_at;9150_at	CLB6;RCK1;PCL9;CLN2;PCL1
GO:0007124	0.00993895	pseudohyphal growth	3989_at;4358_at;4460_i_at;4899_at;5643_at;5655_n_at;6337_at;6453_at;6455_s_at;6459_at;6641_at;7777_at;7859_at;8251_at;9683_at	BI4;SPO16;SAE3;CLB6;RNR1;MEI4;SWI5;ENA5;ENA2;MCD1;PCL9;ECM23;MEI5;SFG1;YOX1
GO:0007126	0.00013919	meiosis	6459_at	MCD1
GO:0007127	0.0016603	meiosis I	4392_at;5609_at;8922_at;9203_at	DSE2;DSE1;SUN4;EGT2
GO:0007131	0.00276299	reciprocal meiotic recombination	4899_at;6641_at;7993_at;9150_at	CLB6;PCL9;CLN2;PCL1
GO:0009145	0.02012023	purine nucleoside triphosphate biosynthetic process	6337_at;6641_at;7777_at;8251_at;9683_at	SWI5;PCL9;ECM23;SFG1;YOX1
GO:0009206	0.02012023	purine ribonucleoside triphosphate biosynthetic process	6337_at;6641_at;9683_at	SWI5;PCL9;YOX1
GO:0009966	0.02012023	regulation of signal transduction	10576_at;10755_at;11079_at;3628_f_at;6453_at;6455_s_at;7546_at;7554_at	PRY2;PIR1;PRY3;COS10;ENA5;ENA2;OPT2;ARR3
GO:0010564	0.0016603	regulation of cell cycle process	4392_at;5609_at	DSE2;DSE1
GO:0010646	0.00276299	regulation of cell communication	6453_at;6455_s_at	ENA5;ENA2
GO:0010876	0.02012023	lipid localization	5609_at;7993_at	DSE1;CLN2
GO:0015672	0.00993895	monovalent inorganic cation transport	8922_at	SUN4
GO:0015850	0.02012023	organic hydroxy compound transport	4358_at;6337_at	SPO16;SWI5
GO:0015918	0.02012023	sterol transport	4899_at;5177_at;6641_at;7993_at;9150_at	CLB6;RCK1;PCL9;CLN2;PCL1
GO:0016049	0.00993895	cell growth	10755_at	PIR1
GO:0016310	0.00194309	phosphorylation	5609_at;7993_at	DSE1;CLN2
GO:0019220	0.000371	regulation of phosphate metabolic process	4460_i_at;6459_at;7859_at	SAE3;MCD1;MEI5
GO:0019236	0.02012023	response to pheromone	10164_at;11301_i_at;4460_i_at;6459_at;7859_at;7981_at;9150_at	TOS4;FLO1;SAE3;MCD1;MEI5;GRE1;PCL1
GO:0022402	1.51E-09	cell cycle process	7204_at	AMN1
GO:0022403	3.59E-10	cell cycle phase	4460_i_at;7859_at	SAE3;MEI5
GO:0022414	0.00445913	reproductive process	6459_at	MCD1
GO:0023051	0.02012023	regulation of signaling	4358_at	SPO16
GO:0030001	0.04025472	metal ion transport	4899_at;6641_at	CLB6;PCL9
GO:0030154	0.00931711	cell differentiation	7993_at	CLN2
GO:0030435	0.04025472	sporulation resulting in formation of a cellular spore	4460_i_at;7859_at	SAE3;MEI5
GO:0030447	0.0016603	filamentous growth	7993_at	CLN2
GO:0031399	0.0016603	regulation of protein modification process	11256_at	IMA5
GO:0032268	0.02691926	regulation of cellular protein metabolic process	6453_at;6455_s_at	ENA5;ENA2
GO:0032502	0.02691926	developmental process	10576_at;11079_at	PRY2;PRY3
GO:0032878	0.02012023	regulation of establishment or maintenance of cell polarity	4358_at;4460_i_at;4796_at;4899_at;5177_at;5609_at;5655_n_at;6337_at;6459_at;6641_at;7204_at;7859_at;7993_at;8251_at;8922_at;9150_at;9203_at;9683_at	SPO16;SAE3;BNS1;CLB6;RCK1;DSE1;MEI4;SWI5;MCD1;PCL9;AMN1;MEI5;CLN2;SFG1;SUN4;PCL1;EGT2;YOX1
GO:0033554	0.00037279	cellular response to stress	11301_i_at	FLO1
GO:0035825	0.00276299	reciprocal DNA recombination	4460_i_at;7859_at	SAE3;MEI5
GO:0040007	0.00081565	growth	6453_at;6455_s_at	ENA5;ENA2
GO:0040020	0.02012023	regulation of meiosis	6459_at	MCD1
GO:0042221	0.01669113	response to chemical stimulus	11256_at	IMA5
GO:0042325	0.000371	regulation of phosphorylation	7204_at	AMN1
GO:0043549	0.000371	regulation of kinase activity	6641_at	PCL9
GO:0043934	0.04025472	sporulation	4899_at	CLB6
GO:0044182	0.0016603	filamentous growth of a population of unicellular organisms	6337_at;6459_at;7204_at;9203_at	SWI5;MCD1;AMN1;EGT2
GO:0044699	5.29E-06	single-organism process	6337_at;9683_at	SWI5;YOX1
GO:0044763	0.0001786	single-organism cellular process	9683_at	YOX1
GO:0044764	0.02235693	multi-organism cellular process	4899_at;6337_at;6459_at;6641_at;7204_at;8251_at;9150_at;9203_at;9683_at	CLB6;SWI5;MCD1;PCL9;AMN1;SFG1;PCL1;EGT2;YOX1
GO:0045003	0.02012023	double-strand break repair via synthesis-dependent strand annealing	3989_at	BI4
GO:0045859	0.000371	regulation of protein kinase activity	4460_i_at;7859_at	SAE3;MEI5
GO:0046034	0.02012023	ATP metabolic process	5609_at	DSE1
GO:0048285	0.00993895	organelle fission	4392_at;5609_at	DSE2;DSE1
GO:0048583	0.00276299	regulation of response to stimulus	7204_at	AMN1
GO:0048610	1.78E-06	cellular process involved in reproduction	5609_at	DSE1
GO:0048646	0.04025472	anatomical structure formation involved in morphogenesis	11256_at	IMA5
GO:0048869	0.01669113	cellular developmental process	8716_s_at	AAD15
GO:0050789	0.02287105	regulation of biological process	3989_at;4358_at;4460_i_at;4899_at;5643_at;5655_n_at;6337_at;6453_at;6455_s_at;6459_at;6641_at;7777_at;7859_at;8251_at;9683_at	BI4;SPO16;SAE3;CLB6;RNR1;MEI4;SWI5;ENA5;ENA2;MCD1;PCL9;ECM23;MEI5;SFG1;YOX1
GO:0050790	0.00445913	regulation of catalytic activity	4899_at;5643_at	CLB6;RNR1
GO:0050794	0.02287105	regulation of cellular process	3989_at;4358_at;4460_i_at;5655_n_at;7859_at	BI4;SPO16;SAE3;MEI4;MEI5
GO:0050896	0.00022598	response to stimulus	3989_at	BI4
GO:0051174	0.000371	regulation of phosphorus metabolic process	5643_at;6453_at;6455_s_at	RNR1;ENA5;ENA2
GO:0051246	0.02691926	regulation of protein metabolic process	6453_at;6455_s_at;7554_at	ENA5;ENA2;ARR3
GO:0051301	2.21E-06	cell division	7546_at	OPT2
GO:0051318	0.00993895	G1 phase	6337_at	SWI5
GO:0051321	0.00013919	meiotic cell cycle	4787_at	TOS2
GO:0051325	0.00026088	interphase	7993_at	CLN2
GO:0051327	0.00013919	M phase of meiotic cell cycle	6455_s_at	ENA2
GO:0051329	0.00026088	interphase of mitotic cell cycle	6337_at	SWI5
GO:0051338	0.000371	regulation of transferase activity	7546_at	OPT2
GO:0051445	0.02012023	regulation of meiotic cell cycle	10164_at;4460_i_at;6459_at;7859_at	TOS4;SAE3;MCD1;MEI5
GO:0051704	0.02235693	multi-organism process	11301_i_at	FLO1
GO:0051716	0.00027892	cellular response to stimulus	6337_at;6641_at;9683_at	SWI5;PCL9;YOX1
GO:0051726	6.67E-05	regulation of cell cycle	3989_at	BI4
GO:0065007	0.03178553	biological regulation	9150_at	PCL1
GO:0065009	0.00445913	regulation of molecular function	7204_at	AMN1
GO:0070783	0.0016603	growth of unicellular organism as a thread of attached cells	3989_at	BI4
GO:0071444	0.02012023	cellular response to pheromone	6337_at;6641_at;7777_at;8251_at;9683_at	SWI5;PCL9;ECM23;SFG1;YOX1
GO:0071900	0.000371	regulation of protein serine/threonine kinase activity	3989_at;4358_at;4460_i_at;4899_at;5643_at;5655_n_at;6337_at;6453_at;6455_s_at;6459_at;6641_at;7777_at;7859_at;8251_at;9683_at	BI4;SPO16;SAE3;CLB6;RNR1;MEI4;SWI5;ENA5;ENA2;MCD1;PCL9;ECM23;MEI5;SFG1;YOX1
GO:2000241	0.000371	regulation of reproductive process	9150_at	PCL1

Cluster 2 -

BP	P_value	GOTerm	Genes	Names
GO:0000460	0.02023891	maturation of 5.8S rRNA	4899_at;6337_at;6459_at;6641_at;7204_at;8251_at;9150_at;9203_at;9683_at	CLB6;SWI5;MCD1;PCL9;AMN1;SFG1;PCL1;EGT2;YOX1
GO:0000462	0.00703327	maturation of SSU-rRNA from tricistronic rRNA transcript (SSU-rRNA, 5.8S rRNA, LSU-rRNA)	6459_at	MCD1
GO:0000466	0.02023891	maturation of 5.8S rRNA from tricistronic rRNA transcript (SSU-rRNA, 5.8S rRNA, LSU-rRNA)	4899_at;6641_at	CLB6;PCL9
GO:0000469	0.04369765	cleavage involved in rRNA processing	5609_at	DSE1
GO:0006139	6.06E-11	nucleobase-containing compound metabolic process	4460_i_at;7859_at	SAE3;MEI5
GO:0006364	1.06E-06	rRNA processing	6453_at;6455_s_at	ENA5;ENA2
GO:0006396	1.39E-07	RNA processing	3989_at	BI4
GO:0006725	4.65E-11	cellular aromatic compound metabolic process	6337_at;9683_at	SWI5;YOX1
GO:0006807	9.07E-12	nitrogen compound metabolic process	6337_at;9150_at;9683_at	SWI5;PCL1;YOX1
GO:0006810	0.00270541	transport	6459_at	MCD1
GO:0006996	0.0104987	organelle organization	7204_at	AMN1
GO:0007049	0.02621421	cell cycle	4392_at;5609_at;8922_at;9203_at	DSE2;DSE1;SUN4;EGT2
GO:0008152	3.64E-11	metabolic process	4358_at;6337_at	SPO16;SWI5
GO:0009058	0.00155379	biosynthetic process	6337_at;6641_at;7777_at;8251_at;9683_at	SWI5;PCL9;ECM23;SFG1;YOX1
GO:0009059	0.01347037	macromolecule biosynthetic process	6337_at;6641_at;7777_at;8251_at;9683_at	SWI5;PCL9;ECM23;SFG1;YOX1
GO:0009987	9.32E-21	cellular process	4358_at;4460_i_at;4796_at;5177_at;5655_n_at;6337_at;6459_at;7204_at;7859_at;9203_at	SPO16;SAE3;BNS1;RCK1;MEI4;SWI5;MCD1;AMN1;MEI5;EGT2
GO:0010467	5.94E-09	gene expression	7993_at	CLN2
GO:0016043	0.00134219	cellular component organization	4392_at;5609_at	DSE2;DSE1
GO:0016070	2.19E-08	RNA metabolic process	4899_at;6641_at;7993_at;9150_at	CLB6;PCL9;CLN2;PCL1
GO:0016072	8.84E-07	rRNA metabolic process	5609_at;7993_at	DSE1;CLN2
GO:0018130	0.02713677	heterocycle biosynthetic process	4460_i_at;7859_at	SAE3;MEI5
GO:0019438	0.02713677	aromatic compound biosynthetic process	11301_i_at	FLO1
GO:0022607	0.02621421	cellular component assembly	5609_at	DSE1
GO:0022613	2.05E-07	ribonucleoprotein complex biogenesis	8716_s_at	AAD15
GO:0030490	0.00703327	maturation of SSU-rRNA	7993_at	CLN2
GO:0034470	2.03E-07	ncRNA processing	7204_at	AMN1
GO:0034622	0.03848755	cellular macromolecular complex assembly	3989_at;4358_at;4460_i_at;4899_at;5643_at;5655_n_at;6337_at;6453_at;6455_s_at;6459_at;6641_at;7777_at;7859_at;8251_at;9683_at	BI4;SPO16;SAE3;CLB6;RNR1;MEI4;SWI5;ENA5;ENA2;MCD1;PCL9;ECM23;MEI5;SFG1;YOX1
GO:0034641	2.73E-11	cellular nitrogen compound metabolic process	6459_at;7204_at;9203_at	MCD1;AMN1;EGT2
GO:0034645	0.01347037	cellular macromolecule biosynthetic process	9683_at	YOX1
GO:0034654	0.02713677	nucleobase-containing compound biosynthetic process	4899_at;6641_at;7993_at;9150_at	CLB6;PCL9;CLN2;PCL1
GO:0034660	1.68E-07	ncRNA metabolic process	4899_at;5177_at;6641_at;7993_at;9150_at	CLB6;RCK1;PCL9;CLN2;PCL1
GO:0042254	3.01E-07	ribosome biogenesis	4899_at	CLB6
GO:0042273	0.03401953	ribosomal large subunit biogenesis	6641_at	PCL9
GO:0042274	0.00310669	ribosomal small subunit biogenesis	4460_i_at;6459_at;7859_at	SAE3;MCD1;MEI5
GO:0043170	3.27E-10	macromolecule metabolic process	6459_at	MCD1
GO:0043933	0.02023891	macromolecular complex subunit organization	4358_at;4460_i_at;4787_at;4796_at;5177_at;5609_at;5655_n_at;6337_at;7859_at;7993_at	SPO16;SAE3;TOS2;BNS1;RCK1;DSE1;MEI4;SWI5;MEI5;CLN2
GO:0044085	6.18E-08	cellular component biogenesis	4460_i_at;7859_at	SAE3;MEI5
GO:0044237	1.27E-10	cellular metabolic process	4899_at	CLB6
GO:0044238	1.27E-11	primary metabolic process	11301_i_at	FLO1
GO:0044249	0.00155379	cellular biosynthetic process	5609_at;7993_at	DSE1;CLN2
GO:0044260	5.49E-10	cellular macromolecule metabolic process	3989_at;4358_at;4460_i_at;5655_n_at;7859_at	BI4;SPO16;SAE3;MEI4;MEI5
GO:0044271	0.02713677	cellular nitrogen compound biosynthetic process	3989_at;4358_at;4460_i_at;4899_at;5643_at;5655_n_at;6337_at;6459_at;7859_at	BI4;SPO16;SAE3;CLB6;RNR1;MEI4;SWI5;MCD1;MEI5
GO:0044281	0.02980997	small molecule metabolic process	4899_at;5643_at	CLB6;RNR1
GO:0044699	7.75E-07	single-organism process	10164_at;10576_at;10755_at;11079_at;11256_at;11301_i_at;3628_f_at;3934_at;3989_at;4358_at;4392_at;4460_i_at;4787_at;4796_at;4899_at;5177_at;5609_at;5643_at;5655_n_at;6337_at;6453_at;6455_s_at;6459_at;6641_at;7204_at;7546_at;7554_at;7777_at;7859_at;7907_at;7912_at;7981_at;7993_at;8251_at;8716_s_at;8922_at;9150_at;9185_at;9203_at;9683_at;9712_at	TOS4;PRY2;PIR1;PRY3;IMA5;FLO1;COS10;RTM1;BI4;SPO16;DSE2;SAE3;TOS2;BNS1;CLB6;RCK1;DSE1;RNR1;MEI4;SWI5;ENA5;ENA2;MCD1;PCL9;AMN1;OPT2;ARR3;ECM23;MEI5;SVS1;AIM44;GRE1;CLN2;SFG1;AAD15;SUN4;PCL1;TOS6;EGT2;YOX1;AIM33
GO:0044763	1.61E-06	single-organism cellular process	4358_at	SPO16
GO:0044765	0.04958734	single-organism transport	8922_at	SUN4
GO:0046483	3.57E-11	heterocycle metabolic process	6337_at;6641_at;9683_at	SWI5;PCL9;YOX1
GO:0050789	0.01919819	regulation of biological process	6337_at;6459_at;7204_at;9203_at	SWI5;MCD1;AMN1;EGT2
GO:0050794	0.01919819	regulation of cellular process	3989_at	BI4
GO:0051179	0.00204782	localization	3989_at	BI4
GO:0051234	0.00270541	establishment of localization	3989_at	BI4
GO:0065003	0.03848755	macromolecular complex assembly	5609_at;7993_at	DSE1;CLN2
GO:0065007	0.01517286	biological regulation	11256_at	IMA5
GO:0071702	0.03848755	organic substance transport	3989_at	BI4
GO:0071704	3.55E-13	organic substance metabolic process	11256_at	IMA5
GO:0071840	1.91E-09	cellular component organization or biogenesis	7993_at	CLN2
GO:0090304	2.40E-09	nucleic acid metabolic process	4460_i_at;7859_at	SAE3;MEI5
GO:0090305	0.02303983	nucleic acid phosphodiester bond hydrolysis	4460_i_at;6459_at;7859_at	SAE3;MCD1;MEI5
GO:0090501	0.04369765	RNA phosphodiester bond hydrolysis	3989_at	BI4
GO:1901360	3.57E-11	organic cyclic compound metabolic process	11256_at	IMA5
GO:1901362	0.02713677	organic cyclic compound biosynthetic process	6337_at;6641_at;9683_at	SWI5;PCL9;YOX1
GO:1901576	0.0017769	organic substance biosynthetic process	6453_at;6455_s_at	ENA5;ENA2

Supplementary Table S6 - Key components in the first segment of diel growth state transition of diatom *Thalassiosira pseudonana*

EntrezGeneID	ProteinID	array_probes	GeneSymbol	Genome	Description
7445065	869	chr1_17784_-,chr1_17987_-,chr1_18127_-		chr_1	PT repeat
7445024	30871	chr1_1019845_-,chr1_1019985_-,chr1_1020188_-		chr_1	Ham1-like protein
7445655	20731	chr1_1031009_+,chr1_1030865_+,chr1_1030725_+		chr_1	mitochondrial carrier protein
7445125	268027	chr1_1510056_-,chr1_1510196_-,chr1_1510336_-		chr_1	Protein of unknown function Met10
7451494	39143	chr1_1636840_+,chr1_1636700_+,chr1_1636560_+	ANT1	chr_1	(ANT1) adenine nucleotide translocator; ATP/ADP translocase
7445530	20952	chr1_2174127_-,chr1_2174267_-,chr1_2174407_-		chr_1	MscS Mechanosensitive ion channel
7449024	14293	chr1_2240238_-,chr1_2240378_-,chr1_2240518_-		chr_1	hypothetical protein
7445006	1855	chr1_2589121_+,chr1_2588981_+,chr1_2588841_+		chr_1	Auxin Efflux Carrier
7447602	2024	chr1_2976676_-,chr1_2976816_-,chr1_2976956_-		chr_1	Protein kinase
7445882	269503	chr10_504420_+,chr10_504260_+,chr10_504120_+	Tp_HSF_1e	chr_10	(Tp_HSF_1e) regulator [Rayko]
7446892	26366	chr10_794586_-,chr10_794726_-,chr10_794946_-		chr_10	oxoglutarate/malate translocator
7450776	36339	chr10_1008761_-,chr10_1008901_-,chr10_1009065_-	ITS1	chr_10	(ITS1) isoleucine-trna synthetase
7450781	24261	chr10_1056514_-,chr10_1056654_-,chr10_1056794_-		chr_10	hypothetical protein
7447991	36958	chr12_753406_-,chr12_753546_-		chr_12	Protein kinase
7447824	26022	chr13_540238_-,chr13_540378_-,chr13_540518_-		chr_13	Protein of unknown function UPF0108
7451616	24757	chr14_61719_+,chr14_61579_+,chr14_61439_+		chr_14	hypothetical protein
7452706	24878	chr14_641679_-,chr14_641819_-,chr14_641959_-		chr_14	DNA-binding SAP
7444306	24977	chr15_243035_-,chr15_243175_-,chr15_243315_-		chr_15	hypothetical protein
7441999	10143	chr15_385140_-,chr15_385280_-,chr15_385420_-		chr_15	Endoplasmic reticulum targeting sequence
7450265	30154	chr15_611196_+,chr15_611056_+,chr15_610916_+	Rpl27	chr_15	(Rpl27) 60S subunit ribosomal protein L27
7450288	10249	chr15_721994_+,chr15_721854_+,chr15_721714_+		chr_15	hypothetical protein
7443996	264350	chr15_913295_-,chr15_913435_-,chr15_913575_-	Tp_CCHH1_Myb2R10	chr_15	(Tp_CCHH1_Myb2R10) regulator [Rayko]
7450326	10542	chr17_15357_+,chr17_15217_+,chr17_15077_+		chr_17	hypothetical protein
7453132	269900	chr17_193040_-,chr17_193180_-,chr17_193320_-	glsF	chr_17	(glsF) glutamate synthase
7444188	10608	chr17_215819_-,chr17_215959_-,chr17_216099_-		chr_17	hypothetical protein
7453139	10612	chr17_224760_+,chr17_224620_+,chr17_224480_+		chr_17	Rhodanese-like
7446272	814	chr19a_157141_-,chr19a_157406_-,chr19a_157618_-		chr_19a_19	hypothetical protein
7449909	25472	chr19a_294084_+,chr19a_293933_+,chr19a_293793_+		chr_19a_19	hypothetical protein
7449916	25478	chr19a_323687_-,chr19a_323827_-,chr19a_323967_-		chr_19a_19	hypothetical protein
7444320	11209	chr19a_451791_-,chr19a_451931_-,chr19a_452071_-		chr_19a_19	hypothetical protein
7444329	25491	chr19a_500256_+,chr19a_500116_+,chr19a_499976_+		chr_19a_19	hypothetical protein
7444342	38272	chr19a_561728_-,chr19a_561868_-,chr19a_562008_-		chr_19a_19	oxidase
7451827	38360	chr19c_70413_+,chr19c_70231_+,chr19c_70091_+		chr_19c_29	Peptidase C1A, papain
7444403	11367	chr19c_175590_+,chr19c_175450_+,chr19c_175310_+		chr_19c_29	hypothetical protein
7448434	2159	chr2_323964_+,chr2_323824_+,chr2_323684_+		chr_2	hypothetical protein
7444630	2435	chr2_1002284_-,chr2_1002424_-,chr2_1002564_-		chr_2	hypothetical protein
7444631	39516	chr2_1004722_-,chr2_1004862_-,chr2_1005002_-		chr_2	Radical SAM
7450382	21311	chr2_1171059_-,chr2_1171199_-,chr2_1171339_-		chr_2	Endoplasmic reticulum targeting sequence
7443052	21363	chr2_1450401_-,chr2_1450541_-,chr2_1450681_-		chr_2	hypothetical protein
7450482	261601	chr2_2505055_+,chr2_2504915_+,chr2_2504775_+		chr_2	Fibronectin, type III subdomain
7452128	14979	chr2_2571662_-,chr2_2571802_-,chr2_2571942_-		chr_2	Aminotransferase, class V
7442414	261613	chr2_2609176_-,chr2_2614871_+,chr2_2609316_-		chr_2	Leucyl/phenylalanyl-tRNA--protein transferase
7442416	3047	chr2_2623747_+,chr2_2623607_+,chr2_2623467_+		chr_2	Endonuclease/exonuclease/phosphatase
7447822	19289	chr20_144634_+,chr20_144494_+,chr20_144354_+	Tp_CBF/NF3	chr_20	(Tp_CBF/NF3) regulator [Rayko]
7443898	11824	chr22_549408_-,chr22_549548_-,chr22_549688_-		chr_22	Glucose inhibited division protein
7444020	32790	chr3_405033_+,chr3_404893_+		chr_3	RNA-binding region RNP-1 (RNA recognition motif)
7449539	261885	chr3_1694422_-,chr3_1694562_-,chr3_1694751_-		chr_3	rna-binding protein
7445385	3895	chr3_2061742_+,chr3_2061602_+,chr3_2061462_+		chr_3	hypothetical protein
7452775	4038	chr3_2408537_+,chr3_2408397_+,chr3_2408257_+		chr_3	C2 calcium/lipid-binding region, CaLB
7446730	4086	chr4_130741_+,chr4_130601_+,chr4_130144_+		chr_4	hypothetical protein
7447505	33312	chr4_740514_+,chr4_740374_+,chr4_740234_+		chr_4	NnrU
7453352	27850	chr4_940570_-,chr4_940710_-,chr4_940850_-	PGAM2	chr_4	(PGAM2) phosphoglycerate mutase
7453353	4408	chr4_942944_+,chr4_942804_+,chr4_942664_+		chr_4	hypothetical protein
7446120	22311	chr4_1346210_+,chr4_1346070_+,chr4_1345930_+		chr_4	Chaperonin Cpn60
7446147	40393	chr4_1498540_-,chr4_1498680_-,chr4_1498820_-	PYK1	chr_4	(PYK1) pyruvate kinase
7446148	22345	chr4_1502053_+,chr4_1501913_+,chr4_1501773_+	PYK2	chr_4	(PYK2) pyruvate kinase
7451969	22430	chr4_1946324_-,chr4_1946464_-,chr4_1946604_-		chr_4	SOUL heme-binding protein
7447253	22535	chr5_151541_+,chr5_151401_+,chr5_151261_+	RS11	chr_5	(RS11) RS11, ribosomal protein 11 40S small ribosomal subunit
7449678	22592	chr5_402473_+,chr5_402333_+,chr5_402128_+		chr_5	CBS
7449701	28239	chr5_488233_-,chr5_488373_-,chr5_488513_-		chr_5	triosephosphate isomerase/glyceraldehyde-3-phosphate dehydrogenase precursor
7446452	22683	chr5_882222_-,chr5_882362_-,chr5_882502_-		chr_5	PT repeat
7450035	22706	chr5_1010654_-,chr5_1010794_-,chr5_1010934_-		chr_5	PPR repeat
7448370	19623	chr6_152776_-,chr6_152916_-		chr_6	RNA-binding region RNP-1 (RNA recognition motif)
7443600	6123	chr6_764014_-,chr6_764154_-,chr6_764294_-		chr_6	Ankyrin
7443620	35041	chr6_864671_+,chr6_864531_+,chr6_864391_+		chr_6	KH
7442377	34730	chr6_1127382_-,chr6_1127522_-,chr6_1127698_-		chr_6	Glucose/ribitol dehydrogenase
7442382	34826	chr6_1166008_+,chr6_1165868_+		chr_6	hypothetical protein
7453272	269141	chr6_1310132_-,chr6_1310272_-,chr6_1310412_-		chr_6	TPR repeat
7453294	6370	chr6_1416239_+,chr6_1416099_+,chr6_1415959_+		chr_6	tRNA/rRNA methyltransferase (SpoU)
7446337	23295	chr6_1811644_+,chr6_1811504_+,chr6_1811364_+		chr_6	Aromatic amino acid permease
7446347	23306	chr6_1878265_+,chr6_1878125_+,chr6_1877985_+		chr_6	hypothetical protein
7446170	23347	chr7_234829_-,chr7_234969_-,chr7_235109_-		chr_7	hypothetical protein
7449192	263129	chr7_675907_+,chr7_675767_+,chr7_675627_+		chr_7	
7449215	23441	chr7_785128_-,chr7_785268_-,chr7_785408_-		chr_7	Patatin
7443420	7338	chr7_1976069_+,chr7_1975929_+,chr7_1975789_+		chr_7	hypothetical protein
7443429	23653	chr8_43300_+,chr8_43160_+,chr8_43020_+		chr_8	Cyclin, N-terminal
7450154	7539	chr8_484955_-,chr8_485095_-,chr8_485235_-		chr_8	hypothetical protein
7452865	29217	chr8_935548_-,chr8_935688_-		chr_8	Ribosomal protein L30e
7444929	23847	chr8_1027959_+,chr8_1027819_+,chr8_1027679_+		chr_8	TraB determinant
7444977	23897	chr9_33068_+,chr9_32928_+,chr9_32788_+		chr_9	hypothetical protein
7443502	7924	chr9_271552_-,chr9_271692_-,chr9_271832_-		chr_9	hypothetical protein
7451005	18076	chr9_1112400_+,chr9_1112222_+,chr9_1112082_+		chr_9	Adenylate kinase
